# ADFIST: Adaptive Dynamic Fuzzy Inference System Tree Driven by Optimized Knowledge Base for Indoor Air Quality Assessment

**DOI:** 10.3390/s22031008

**Published:** 2022-01-28

**Authors:** Jagriti Saini, Maitreyee Dutta, Gonçalo Marques

**Affiliations:** 1 National Institute of Technical Teacher’s Training and Research, Chandigarh 160019, India; d_maitreyee@yahoo.co.in; 2 ESTGOH, Polytechnic of Coimbra, Rua General Santos Costa, 3400-124 Oliveira do Hospital, Portugal

**Keywords:** indoor air quality, fuzzy inference system, pollution, optimization, public health

## Abstract

Air quality levels do not just affect climate change; rather, it leaves a significant impact on public health and wellbeing. Indoor air pollution is the major contributor to increased mortality and morbidity rates. This paper is focused on the assessment of indoor air quality based on several important pollutants (PM_10_, PM_2.5_, CO_2_, CO, tVOC, and NO_2_). These pollutants are responsible for potential health issues, including respiratory disease, central nervous system dysfunction, cardiovascular disease, and cancer. The pollutant concentrations were measured from a rural site in India using an Internet of Things-based sensor system. An Adaptive Dynamic Fuzzy Inference System Tree was implemented to process the field variables. The knowledge base for the proposed model was designed using a global optimization algorithm. However, the model was tuned using a local search algorithm to achieve enhanced prediction performance. The proposed model gives normalized root mean square error of 0.6679, 0.6218, 0.1077, 0.2585, 0.0667 and 0.0635 for PM_10_, PM_2.5_, CO_2_, CO, tVOC, and NO_2_, respectively. This approach was compared with the existing studies in the literature, and the approach was also validated against the online benchmark dataset.

## 1. Introduction

The Environmental Protection Agency (EPA) defines indoor air quality (IAQ) as the quality of air within building premises or closed rooms that can leave a significant impact on occupant health, comfort, and productivity levels [1]. It is already proven that human activities, industrial operations, and increasing traffic on roads are the major factors behind the deterioration in the environment [2]. Along with several outdoor sources, IAQ is affected by inadequate thermal comfort levels due to high humidity and temperature in the closed structures, inadequate ventilation management, hazardous building materials, and day-to-day human activities [3]. The rising concentrations of harmful pollutants within indoor environments is further linked to the deteriorating health of building occupants, especially elderly, infants, persons with disabilities, and household women, as they spend most of their time indoors [4,5,6]. Therefore, it is crucial to understand all aspects of indoor air pollution (IAP) and its impact on public health, while identifying potential solutions for IAQ management in the closed structures. 

As per a survey representing almost 30,000 institutions from the United States, it was observed that environmental air quality control is the prime requirement of the country as it is causing major damage to the health and wellbeing of the people [7]. IAP is directly associated with rising cases of morbidity and mortality in both developed and developing countries, as it is directly linked to several acute and chronic diseases [4,8]. The short-term and long-term health effects are reported depending upon the level and duration of exposure to harmful pollutants. The symptoms linked to short-term health effects include wheezing, eye/skin irritation, and nasal congestion; however, they are preventable [9]. On the other side, long-term health effects are respiratory infections [10], pulmonary tuberculosis [11], adverse pregnancy outcomes [12], asthma [13], chronic bronchitis [14], cancer [15], and heart disease [16]; the patients may report them after repeated and long periods of exposure. 

There are a variety of pollutants that affect the health of building occupants inside homes, offices, cafes, schools, hospitals, and shopping malls. Due to the increasing transportation activities, industrial activities, and infrastructure, the ambient air pollution levels are increasing with a sharp curve, which ultimately accounts for the decaying IAQ levels as well [17]. Other than this, there are several potential sources of pollutants in the indoor environment at rural and urban buildings. Rural homes experience decay in air quality levels due to repeated use of coal, cow dung, wood, and kerosene for heating and cooking purposes [5]. Unventilated homes in urban areas reduce the circulation of healthy air for breathing [18]. The list of harmful pollutants that affect the building environment includes particulate matter (PM), carbon dioxide (CO_2_), carbon monoxide (CO), sulphur dioxide (SO_2_), nitrogen oxides (NO_x_), volatile organic compounds (VOCs), ozone, radon, heavy metals, bioaerosols, and antioxidants [3]. 

IAQ management is critical for better public health outcomes. Therefore, field experts and researchers from the past several years are working in this direction to identify reliable solutions. Numerous researchers are using the Internet of Things (IoT) and Wireless Sensor Network (WSN) based technologies to design IAQ monitoring solutions to assess air quality in the building premises. Xie et al. [19] designed an artificial neural network (ANN) based IAQ prediction system while focusing on six IAQ variables and three thermal comfort parameters. They used occupant symptom metric to measure IAQ levels. Backpropagation based feed-forward network with variable learning rate and momentum term was used for ANN modelling. Tagliabue et al. [20] designed an IoT based system to gather IAQ data from educational building at University of Brescia. The main goal of this work was to regulate the HVAC system along with the opening and closing patterns of lab windows to enhance IAQ levels in the premises that could further enhance the learning performance of occupants. The proposed methodology suggests efficient use of ANN for triggering enhanced ventilation rate control via IoT communication protocol. Ahn et al. [21] used deep learning based models with IAQ sensor data for estimating atmospheric changes. They used two deep learning methods: gated recurrent unit (GRU) and long-short term memory (LSTM) network for time series data analysis. The optimal time step search approach proposed by authors in this study presented best learning performance in comparison to conventional models. 

The authors have already published comprehensive [22] and systematic reviews [23,24] focusing on existing advancements in the field of IAQ monitoring and assessment while highlighting the need, challenges, and future scopes in this important field of research to provide insights to the related work in this field. Other than this, several studies have also been published on the development of prediction systems based on neural networks, machine learning, and deep learning approaches to assist building occupants with a prior indication of harmful pollutant concentrations. Furthermore, the authors have published a systematic review to highlight the contribution of existing researchers in the field along with the gaps in literature [25].

The main objective of this paper is to present an Adaptive Dynamic Fuzzy Inference System Tree (ADFIST) based approach to predict potential indoor air pollutants from the target sites. The authors have collected real-time data from four different rural and urban sites on six important IAQ parameters, including PM_10_, PM_25_, CO_2_, CO, NO_2_, tVOCs, along with two crucial thermal comfort parameters—temperature and humidity. An approach consisting of aggregating multiple fuzzy inference systems (FIS) with a specific set of inputs considering their correlation with the response variable was used, and the rule learning process was further optimized using particle swarm optimization (PSO). Ultimately, the model was tuned using a pattern search algorithm to achieve enhanced prediction accuracy so that the proposed system can be implemented in real-time scenarios to avoid critical consequences associated with poor IAQ levels. The proposed model is called dynamic because it uses a dynamic combination of input features as per the selected response variable for prediction. The adaptive nature of the model corresponds to its rule learning behaviour which is optimized by PSO and Pattern Search to ensure a unique set of rules at every stage for different input–output combinations. 

The performance of the proposed method was analysed through normalized root-mean-square error (NRMSE), normalized mean square error (NMSE), coefficient of determination (R^2^), and mean absolute percentage error (MAPE). In this paper, Section 2 provides details about the experimental design of an IoT-based monitoring system, real-time parameter collection from the field, and data pre-processing; Section 3 includes detailed information about methodology and methods. Furthermore, Section 4 presents the results and discussion about model performance in terms of the above-mentioned performance indicators, and finally, the conclusion is presented in Section 5. 

## 2. Materials and Methods

**Figure 1:**
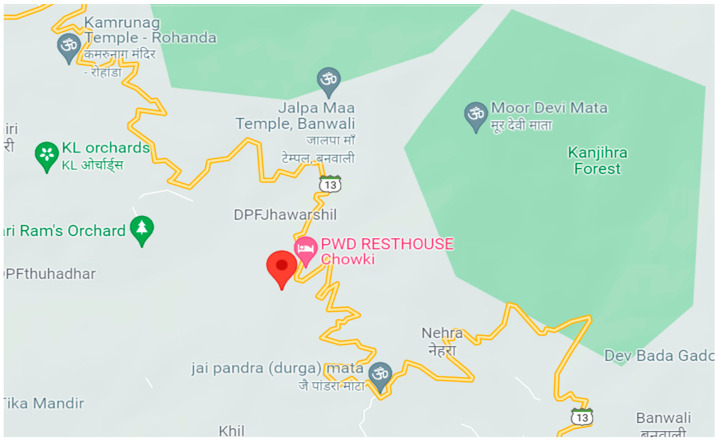
Monitoring site location displayed using Google Maps.

Several primary and secondary pollutants affect IAQ levels. However, their concentration varies depending upon the geographical area under consideration. The authors focused on collecting real-time data on potential pollutants from a rural site in India. The preferred IAQ parameters for this study were PM_10_, PM_2.5_, CO_2_, CO, NO_2_, tVOCs, and along with these, two major thermal comfort parameters were also monitored: temperature and humidity. These eight parameters were selected out of many other crucial IAQ and thermal comfort parameters only after studying the environmental conditions of the target geographical area. Recommendations from air pollution experts in the area were also taken regarding the selection of parameters to address the field IAQ concerns. The hardware monitoring system was installed in the kitchen area of a home located in a rural village of Himachal Pradesh, where traditional heating and cooking practices are followed. The map of the selected monitoring site is provided in Figure 1. The major sources of pollution at this site are the fireplace that involves the burning of wood, cow dung, kerosene, dry grass, and traditional cooking practices. The detailed information on monitoring system design and parameter collection is provided in the subsections below. 

### 2.1. Monitoring System Design

The design of the proposed IAQ monitoring system was based on IoT technologies. The authors used four different IAQ sensor modules along with an Arduino Uno microcontroller and ESP8266 communication module to collect information on concentration, eight different focus IAQ, and thermal comfort parameters. CCS811 sensor was preferred for measuring CO_2_ and tVOC concentration, SDS011 sensor provided field data on PM_10_ and PM_2.5_ parameters; whereas Grove—Air quality sensor v1.3—MP503 module was used to measure the concentration of NO_2_ and CO from target sites. Other than this, the DHT11 sensor module was used for measuring thermal comfort parameters (temperature and humidity). Table 1 provides manufacturer specifications for all these sensor modules. The above-mentioned sensors were connected to the Arduino Uno microcontroller for collecting real-time data from the target field environment. Furthermore, the ESP8266 module was used as a gateway to transfer field data to the centralized online platform. ThingSpeak open-source platform was used for collecting real-time data from the target rural site. Numerous competitive sensor modules in the market can be used to create IoT-based IAQ monitoring systems. The main focus of the authors was to design a cost-effective system while ensuring calibrated data collection. The overall cost of a complete hardware monitoring system in this study turned out to be USD 81.60, including the estimated cost of Arduino Uno (USD 6.73), ESP8266 gateway unit (USD 1.35), and miscellaneous items such as wires and breadboards (USD 3.37).

**Figure 2:**
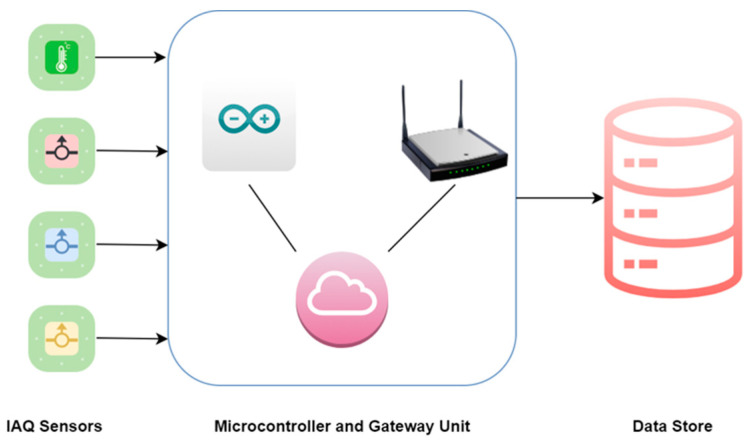
Hardware monitoring system architecture.

The authors used factory-calibrated sensor units to ensure reliable real-time data collection from the field environment. However, the authors also preferred conducting field reliability tests before deploying sensors in the field. For the DHT11 sensor, the reliability was tested in the laboratory against a standard instrument (Honeywell TM00X), whereas for the remaining three sensors, the manufacturer specified procedures were conducted to ensure stable performance. The hardware monitoring system was set to provide readings on-field pollutant concentrations after every 5 min, and the total duration of measurements was six months from 1 January 2021 to 30 June 2021. The general architecture of IoT based IAQ monitoring system is given in Figure 2. 

### 2.2. Data Pre-Processing

The real-time data stored on the ThingSpeak channel was exported in the form of an excel file. It was expected to receive 51,840 samples from the field within the given duration of 6 months. However, due to system failures, maintenance issues, and other field-associated errors, a total of 42,051 samples were recorded with an 18.8% error rate. The dataset containing eight different IAQ and thermal comfort parameters was first analyzed using boxplots Figure S1 (Supplementary Materials), and considerable amounts of samples were found outside the lower and upper quartile ranges. The samples beyond the outer fence of the boxplot are called extreme outliers, and they are required to be removed to increase statistical power and to reduce variability in data. Considering the recommendations provided by existing researchers in the literature, authors preferred using the Interquartile range method for outlier removal [26,27]. In this method, Q1 (first quartile) represents the 25 percentiles, cutting off the lowest 25% of data, and Q3 (third quartile) cuts off the highest 25% of the data while giving the intermediate values of the data distribution at the output. The formula for extracting the middle half of data while removing outliers above and below the quartile range is given by Equation (1) [28].

(1) IQR=Q3 – Q1

After outlier removal, the data containing 30436 samples was processed further to test the presence of missing values. The dataset had 22.3% missing values. As field data does not contain nominal attributes, the authors decided to use the mean imputation method for missing value imputation [29,30]. In this method, the missing data cells are filled with the mean value of the respective attribute. As the data samples from the field are collected every 5 min, it is necessary to use a standard averaging method to summarise data readings. Therefore, the next step in the data pre-processing was to apply mean-hour reduction, which provided 3695 samples at the output. The statistical information in terms of mean, std, min, max, and quartile ranges of the dataset is presented in Table 2. These samples were further utilized for prediction model design.

**Figure 3:**
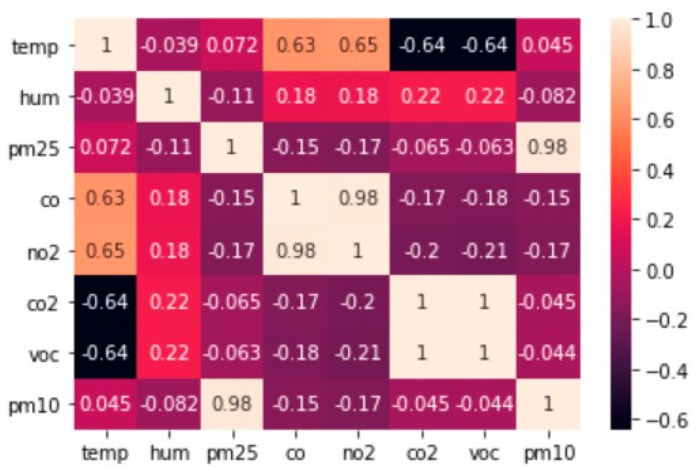
Pearson correlation between parameters.

The literature states that environmental data are random in nature due to cyclic variations, seasonal variations, and irregular movements [31,32,33]. Other than this, due to the use of low-cost sensors, a considerable amount of measurement uncertainty and variability is also introduced to the field data [34]. Therefore, the authors decided to use a FIS to process the field data that is fuzzy in nature. The data was analysed on the basis of correlation between features. Figure 3 shows a correlation plot based on the Pearson Correlation method to describe the general distribution of data. The information obtained from correlation analysis will be further utilized in designing DFIST for IAQ prediction. After analysing the correlation response of variables, the authors used the k-fold cross-validation approach for data splitting, as suggested by [35,36]. The Naïve-Bayes based cross-validation was used to split data with 20% holdout on a total of 3695 samples; as a result, 2956 samples were left for training data and 739 for validation data [37,38,39]. The available dataset was further utilized for prediction model training and validation performance assessment. 

### 2.3. Parameter Classifications

The Environment Pollution Agency (EPA) has already defined air quality levels based on their negative impact on public health. We followed EPA guidelines to identify and evaluate the IAQ parameters according to their safe and unsafe limits. Based on the available information in the literature. Consequently, the IAQ parameter ranges were defined using five different categories as follows:

- Good: This is considered appropriate to perform normal day-to-day activities.
- Moderate: Indoor activities can be performed; however, children and elderly people may be affected.
- Unhealthy: Indoor activities must be avoided; especially for children and adults with respiratory health issues.
- Poor: Sensitive groups may experience serious discomfort. In this situation, it is necessary to implement pollution emission controlling measures on a priority basis.
- Hazardous: Recommendations for following serious measures to protect the health of the building occupants by using adequate ventilation and air quality purification measures.

These threshold levels are further used in model training to help to make decisions about the forecasting values for the respective response variables. 

## 3. Methods

Decaying IAQ levels in the residential and commercial buildings is a matter of concern for occupant health and wellbeing. Adequate technological interventions can ensure promising solutions for IAP management and control. In Section 2, details about the development of IoT based monitoring system has been discussed. It is further possible to integrate the potential of Artificial Intelligence (AI) to perform real-time assessment and control of environmental factors. As data collected from the field environment has higher variability and is fuzzy in nature, authors preferred using the potential of fuzzy systems to design a prediction system to address the concerns. The concept of fuzzy logic was first proposed by Lotfy A. Zadeh in the mid-1960s [40]. A fuzzy set is defined as the class of objects having different degrees of membership, and each set in fuzzy logic systems is characterized by a unique set of membership functions. The degree of membership for each object in fuzzy systems can range between 0 and 1. The main advantage of fuzzy logic is that the concept is influenced by human reasoning and the degree of belongingness of object to the class is measured in proportions. There are four main elements of a fuzzy logic architecture: fuzzification, inference engine, rule base, and defuzzification:

- The Fuzzification step converts crisp inputs into fuzzy systems. These crisp inputs are generally the data measured by sensors that are required to be passed to a fuzzy control system for further processing.
- Rule base plays an important role in fuzzy decision-making. Rule base or knowledge base is basically a set of in-then conditions developed from expert knowledge or field conditions.
- The inference engine defines the degree of match between fuzzy input and knowledge base. It decides which rules must be implemented to achieve the desired output as per the given input.
- Defuzzification is the process of converting fuzzy sets back into crisp values that can be further used in real-life environments.

A simple fuzzy logic system is required to have m^n^ number of rules; where m = the number of membership functions assigned per input variable and n = the total number of inputs [41,42]. The available field IAQ data have seven input variables and one output variable. For instance, on assigning five membership functions per input variable to depict EPA-based pollutant concentration levels, the system may need to have 5^(7)^ = 78,125 number of rules. It is quite difficult to design such a huge number of fuzzy rules manually and the process is prone to errors. Furthermore, a system with such a large number of rules is likely to have high computational complexity. Moreover, the practical realization of such a system is challenging. Therefore, the authors decided to use the hierarchical structure for fuzzy system design to forecast IAQ response variables [43,44]. FIS can provide good reasoning for the pollutant concentrations while processing field variables with their unique scales. The hierarchical structure of FIS makes use of km^n^ number of rules; where k = number of FIS used in the system design, m = number of membership functions per input variable, and n = number of input variables [41,42]. In this tree form, every FIS module takes two inputs only, and numbers of FIS modules are arranged in a cascaded manner to accommodate all input variables for obtaining final forecasting outcomes. Therefore, the total number of rules required for designing a hierarchal FIS structure with seven input variables, five membership functions per input, and six FIS modules to accommodate 7 inputs at different levels will be 6 × 5^(2)^ = 150 only. Therefore, the computational complexity and design issues for FIS based forecasting system can be resolved with ease. Furthermore, the hierarchal approach helps to evaluate input features based on their correlation with the response variables so that the prediction accuracy can be enhanced. In the second phase, PSO has been utilized to optimize the rule learning process, and in the final phase, the network is tuned using a pattern search algorithm. The entire process is explained in detail in the below sub-sections.

### 3.1. Fuzzy Inference System Tree

FIS makes use of fuzzy logic to design an expert system that can perform a reasoning process with the mapping of several input vectors to a single output. There are two different types of FIS: Mamdani and Sugeno. For the proposed study, the authors used Mamdani FIS due to its simplest structure and enhanced freedom to map antecedents to consequents with fuzzy membership [45,46]. In the first phase, an input assessment model is designed using fuzzy membership functions. The pollutant concentration levels are evaluated based on their impact on human health, and the ranges are divided into five different categories as defined in Section 2.3. However, the thermal comfort parameters (temperature and humidity) are defined using three different categories: good/low, moderate, and high. In this work, authors used triangular membership functions for defining parameter ranges recommended by [47] to transform respective input real-valued parameter ranges into fuzzy values ranging between 0 and 1. The mathematical expression for triangular membership functions is defined in Equation (2).

(2) $$\mu \left(x,\;a,\;b,\;c\right)=\max\begin{array}{c} \left\{\min\left\{\frac{x-a}{b-a},\frac{c-x}{c-b}\right\},\;0\right\} \end{array}$$

where *x* = input pollutant concentration, *a*, *b*, *c* are parameter ranges defined by membership functions. They usually vary as per the defined limits of the respective pollutants under EPA guidelines. After assigning membership functions to each concentration level, the reasoning process can be implemented with the help of specific operators. Before developing FIS, it is necessary to develop an understanding of fuzzy operators used in the reasoning process. In this work, the authors have used two main fuzzy operators: Union and Intersection, which represent OR & AND operation, respectively [48]. The mathematical representations of these operators are given in Equations (3) and (4).

(3) $$\mathrm{Union}\;(\mathrm{OR}):\;\mu_{A\cup B}\left(x\right)=\max\left\{\mu_{A}\left(x\right),\;\mu_{B}\left(x\right)\right\}$$

(4) $$\mathrm{Intersection}\;(\mathrm{AND}):\;\mu_{A\cap B}\left(x\right)=\min\;\left\{\mu_{A}\left(x\right),\;\mu_{B}\left(x\right)\right\}$$

In terms of IAQ assessment, FIS models are more useful due to their subjectivity handling [40]. The fuzzy logics make it easier to interpret the existing knowledge while mapping to a level of uncertainty to specific evaluations on the fuzzy scale. To ensure accurate prediction of parameter concentrations, it is necessary to access all IAQ scenarios carefully while presenting hazardous concentrations with accurate descriptions. Fuzzy reasoning provides better opportunities to address pollutant concentrations with their unique impact on human health. The knowledge/rule base can be designed accordingly as per expert guidelines on air quality situations. For instance, if the temperature is low and PM_2.5_ is good, then PM_10_ is good. Different parameter concentrations can be linked to each other based on the correlation to each other, guidelines provided by air quality experts, and the studies already published in the literature [49,50]. The fuzzy reasoning for different pollutant concentration levels, represented by different membership function ranges, can be given as below:

Sample Rule 1: If Temp is low and PM_2.5_ is good, then PM_10_ is good.

Sample Rule 2: If Temp is moderate and PM_2.5_ is moderate, PM_10_ is moderate.

The decision regarding the impact of rule antecedent combination on consequent conditions is made on the basis of existing research and expert guidelines [40,51,52]. When the rule contains the AND operator between different antecedent parameters, the combination can be evaluated jointly using the min operator. For example, if the fuzzy value for temperature low is equal to 0.1 and for PM_2.5_ moderate is 0.3, then the min operator for this condition with PM_10,_ can, consequently, be presented by Equation (5).

(5) $$\mu_{PM10}=\;\min\left\{0.1,\;0.3\right\}=0.1$$

**Figure 4:**
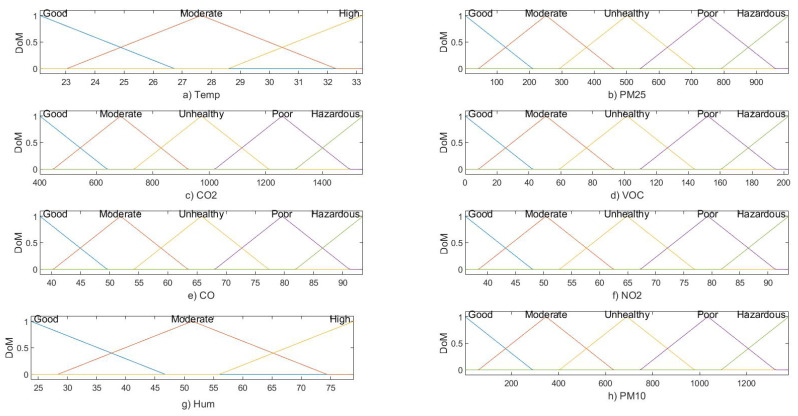
Membership functions for air quality parameters: (**a**) Temp, (**b**) PM_2.5_, (**c**) CO_2_, (**d**) tVOC, (**e**) CO, (**f**) NO_2_, (**g**) Hum and (**h**) PM_10_.

The rule base is further aggregated using the max operator to receive output value [48]. After computing the individual rules, the superimposed area of all rule outputs indicates the final outcome of the reasoning-based evaluations [48]. The membership functions for input features and with PM_10_ as response variable are shown in Figure 4. At the final stage of the FIS, a defuzzification method is used to obtain real-time outputs for the field-based study. The defuzzification method used in this study is Centroid Function, and the formula for calculating defuzzified values out of aggregated values is given in Equation (6) [53]. This method provides the centre of the area under the curve as per the output membership function [54]. This method returns a value using the restrictions defined with input membership functions ranging from good to hazardous.

(6) $$x^{*}=\frac{\int^{\;}\mu_{c_{m}}\left(x\right)\cdot \;x^{\prime }dx}{\int^{\;}\mu_{c_{m}}\left(x\right)\;dx\;}$$

The system robustness usually depends upon the number of rules in the FIS. However, instead of designing the rules manually for all input parameters, the authors used PSO to the optimized rule learning process.

### 3.2. Particle Swarm Optimization

**Figure 5:**
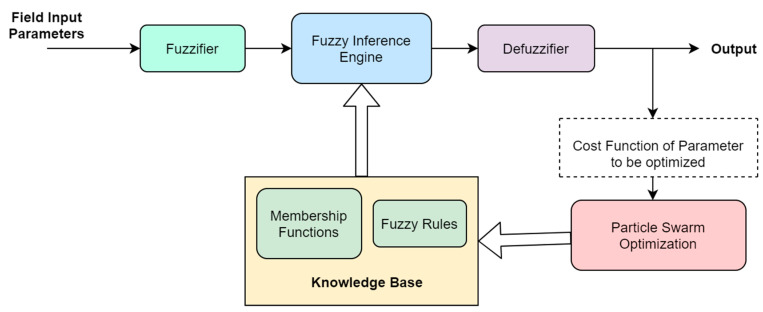
General flow of FIS system with PSO rule learning.

PSO is a global optimization algorithm that is inspired by swarm intelligence [55]. In this approach, the particle is used to indicate the swarm with the two necessary parameters: position and velocity [56]. All particles in the group work over the same principles, under similar working conditions, to find the best personal and best overall fitness values. The objective function is usually problem-dependent, and several iterations are performed to achieve good results for the target problem space [57]. Each particle has a specific velocity and position vector in the problem space, and it is updated after every learning cycle to achieve the best results. The new velocity and position values are obtained as per Equations (7) and (8), respectively [56].

(7) $$V_{i}\left(t+1\right)=wV_{i}\left(t\right)+c_{1}r_{1}\;\left(P_{pbest}\left(t\right)-P_{i}\left(t\right)\right)+c_{2}r_{2}\left(P_{gbest}\left(t\right)-P_{i}\left(t\right)\right)$$

(8) $$P_{i}\;\left(t+1\right)=P_{i}\left(t\right)+V_{i}\left(t+1\right)$$

where *P_i_* represents position vector and *V_i_* represents velocity vector in the problem space. Furthermore, *c*_1_ and *c*_2_ are acceleration constants, *r*_1_ and *r*_2_ are random numbers distributed between 0 and 1. Inertia weight (*w*) is utilized as a control parameter to adjust the impact of prior velocity values on the current velocity value [56]. As PSO is a global search algorithm, it has the ability to produce a relevant knowledge base for the FIS when employed at the rule learning stage. The general algorithm for PSO can be given in Algorithm S1 (Supplementary Materials) [55]:

For the given problem, the parameter initialization was carried out as follows: *w* = 1, *c*_1_ = 1, *c*_2_ = 2, population size = 100 and the number of iterations at PSO rule learning stage were kept 150. To optimize the rule learning process for the FIS system, the authors first designed the hierarchical structure of FIS to process input features as per their correlation with the response variable. Consequently, the membership functions were defined as per the air quality concentration guidelines, and the values were assigned to individual parameters. Finally, the FIS system was allowed to learn rules with the PSO algorithm. For the current problem, each particle is adjusted to achieve model rules corresponding to input membership functions. Each particle represents the potential best solution, and it is updated every time using Equations (7) and (8) to achieve global best fitness value. Figure 5 represents the general flow of the FIS system with PSO as a knowledge base optimization algorithm.

### 3.3. Pattern Search Algorithm

Pattern search or direct search is a derivative-free search algorithm that belongs to the numerical optimization methods [58]. It does not require a gradient to achieve the best value for the objective function. It is widely used for identifying the best match or the solution with the lowest error value in the problem space. This method is widely used for multidimensional analysis [59]. For the current experimental analysis, the pattern search algorithm is applied at the final model tuning stage to enhance the prediction accuracy. The number of iterations at this stage was kept 100, and the focus tuning parameters were rule base, inputs, and the model output. The general steps for pattern search algorithm are given in Algorithm S2 (Supplementary Materials) [58]. This algorithm works with an initial guess at solution x_0,_ and the initial choice for the step length parameter is generally Δ_0_ > 0.

### 3.4. ADFIST Implementation

**Figure 6:**
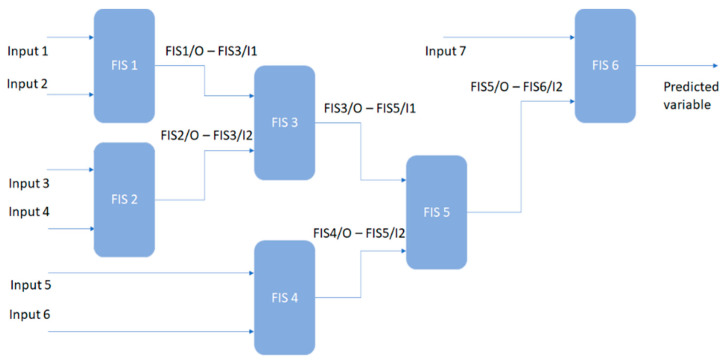
General architecture of DFIST model.

To design the proposed ADFIST model, the authors arranged multiple FIS in the form of an incremental tree. As the ADFIST is desired to provide a prediction for all IAQ parameters, a dynamic approach was followed for setting up input and output variables for particular instances. As there are seven input features, we have aggregated six FIS systems to accommodate all inputs; however, two inputs were given at one stage. The reason to use only two input and single output FIS per stage is to limit the number of rules that could further reduce the computational complexity at every stage [60]. The decision about the organization of input parameters at every stage was made based on the correlation between features and the response variable, as shown in Figure 3 [61,62,63]. In the first stage, the correlation between the response variable and all input features was tested, and then the correlation between features was analyzed. The general structure of DFIST is given in Figure 6 with 6 FIS at different stages, feeding two input parameters to one FIS module.

For instance, when PM_10_ was selected as a response variable, among all input features, PM_2.5_ presented maximum correlation (0.98). However, among thermal comfort parameters, temperature provided a positive correlation of 0.045. Therefore, these two features were used at the first stage of the FIST and were fed to FIS1. For the next stage, as we have all features with negative correlation, the combination for FIS2 was decided based on the features that show the highest correlation with each other. Therefore, tVOC and CO_2_ were connected to FIS2, and then the output of FIS1 and FIS2 were combined at FIS3. Furthermore, FIS4 was fed with CO and NO_2_ as they show the highest correlation with each other. The output of FIS3 and FIS4 was combined at FIS5, whose output is further given to FIS6 as the second input, and the first input is given in the form of one remaining thermal comfort parameter (humidity). Finally, the FIS6 provides the desired output of the DFIST, which is considered as a prediction output. Furthermore, the DFIST is made adaptive to the changing input and output combinations with the help of two important optimization algorithms. Therefore, the proposed model is named as Adaptive Dynamic Fuzzy Inference System Tree—ADFIST.

**Figure 7:**
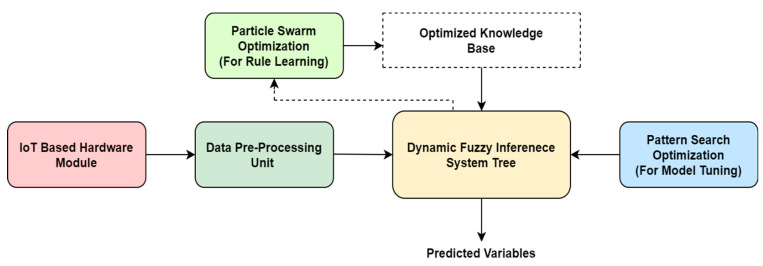
Full system architecture for proposed ADFIST IAQ parameter prediction approach.

The adaptive behaviour of the model is reported at every stage of the tree as the PSO algorithm is used with every FIS module to optimize the rule learning process with every dynamic combination of input features. The pattern search algorithm is further used to tune the entire system for enhanced prediction performance. The complete architecture of the proposed ADFIST model is given in Figure 7. The full list of rules (in the case of PM_10_ prediction) for ADFIST is provided in Supplementary Materials.

The model performance was further evaluated in terms of NRMSE, NMSE, R^2,^ and MAPE. NRMSE facilitates the assessment of models that work on a dataset with variable scales. It is preferably expressed in percentage and the lower value is usually better as it represents lesser residual variance. The mathematical formula for NRMSE is given in Equation (9). In a similar manner, NMSE is selected for evaluation because of its ability to avoid bias towards models that over-predict or under-predict. As the model accuracy is inversely proportional to NMSE, lower value links to better model performance. The mathematical formula for NMSE is given in Equation (10). Furthermore, R^2^ is another widely recommended statistical measure to assess the strength of the linear relationship between variables. The formula for the R^2^ parameter is given in Equation (11); the higher value of this parameter is expected. MAPE is defined as the difference between measured and forecasted values. It is given in percentage and the smaller value of MAPE indicates better forecasts. The mathematical formula for MAPE is given in Equation (12) [64,65].

(9) $$NRMSE=\frac{\sqrt{\frac{\sum_{i=1}^{N}{\left(P_{i}-O_{i}\right)}^{2}}{N}}}{ℳ}$$

where *P_i_* and *O_i_* are predictions and observations, respectively; *N* represents a number of observations, and ℳ is the mean of the observations.

(10) $$NMSE=\frac{\bar{{\left(C_{o}-C_{p}\right)}^{2}}}{\bar{C_{o}}\;\bar{C_{p}}}$$

where *C_o_* and *C_p_* present observed and predicted concentrations, respectively, and the overbar indicates mean over the data sampling points.

(11) $$R^{2}=\frac{MSS}{TSS}=\;\frac{TSS-RSS}{TSS}$$

where *MSS* represents the model sum of squares, and it can be given as the sum of squares of forecasts from linear equation minus mean of that respective variable. *TSS* represents the total sum of squares associated with the respective outcome variable.

(12) $$MAPE=\frac{1}{n}\;\overset{n}{\underset{t=1}{\sum }}\left|\;\frac{A_{t}-F_{t}}{A_{t}}\right|$$

where *n* is the number of fitted points, *A_t_* is the actual value, and *F_t_* is the forecast value.

## 4. Results and Discussions

The design methodology and the approach for implementation of ADFIST are already discussed in Section 3. The proposed model was trained using real-time data obtained from the IoT-based hardware module installed at a rural site. The pre-processing data stages are also explained in Section 2. After obtaining detailed information on field data, and feature correlation, the ADFIST system was fed with the respective input features based on the selection of response variable at a particular instance. The combination of seven external input features and the response variables is given in Table 3.

As shown in Figure 6, the output of FIS1 and FIS2 was combined to fed FIS3, and outputs of FIS3 and FIS4 were further fed to FIS5; however, the output of FIS5 was fed to the second input of FIS6. As the combination of input features and the hierarchy varies as per the selected response variable, the proposed model shows dynamic behaviour to meet the specific requirements of the end-users. For instance, when the end-user requires the model to predict PM_10_, all the inputs at different levels of the trees will be adjusted as per row 3 of Table 3. As described in the data pre-processing, the model was trained using 2956 samples; however, 739 (20%) random samples were kept for model validation.

The proposed ADFIST model is trained using pre-processed data samples. The performance of a fuzzy system is highly dependent on the knowledge base. However, the biggest challenge for researchers during experimental studies is to design the most accurate and responsive rule base [66,67]. An incorrectly designed rule base can have a negative impact on model performance [68]. Therefore, instead of designing the rule base of the proposed ADFIST model manually, the authors used PSO for rule learning. Generally, the formation of rules in FIS systems is based on the number of variables and the number of assigned membership functions. The higher system complexity due to the higher number of rules at every stage makes it difficult to implement and realize FIS models. The system complexity further increases with the higher number of input features. Moreover, the structure may also become complicated when FIS are connected in tree form to process inputs on the basis of their priority at different stages.

The global search optimization algorithms can be useful to handle such model complexities [69]. PSO identifies the optimal regions of available complex search spaces by interacting with every individual in the particle population [56]. Moreover, PSO is known to present a high-quality solution in short computation time, and the convergence characteristics are also better than other stochastic approaches available in the literature [69]. PSO enables the realization of a knowledge base simpler with a limited number of parameters and an easy implementation strategy. Therefore, the authors used PSO for optimizing the rule learning process at every stage of the proposed ADFIST system. Moreover, the model adapts to the new rule base as per the selected response variable and the combination of input features. This adaptive behaviour makes it a reliable solution for changing parameter conditions and combinations in different scenarios. The adaptive nature of the knowledge base with changing input combinations and arrangements as per selected response variable is shown in Table 4.

For the prediction of PM_10_, the proposed ADFIST model used a total of 111 rules; however, for PM_2.5_, CO_2_, tVOC, CO, and NO_2_, the total number of rules were 99, 118, 113, 113, and 115, respectively. The PSO at the first stage helped to optimize rule learning as per the correlation-based input parameter combinations. However, pattern search at the second stage tuned ADFIST performance based on the input-output combinations while eliminating redundant rules to improve computation time and cost of the system. The numbers of iterations were set at 150 for the PSO rule learning stage and 100 for the Pattern Search-based model turning stage. However, the ADFIST model converged at different levels due to the fast convergence performance of PSO with a unique set of input–output combinations and rules. The ADFIST model performance is measured in terms of four essential performance indicators: NRMSE, NMSE, R^2^, and MAPE [70,71,72]. The main reason behind the selection of NRMSE and NMSE for model evaluation is that field data includes multiple parameters with different units and varying ranges [73,74,75]. RMSE is a scale-dependent performance indicator. Consequently, it is not possible to generalize model performance using this indicator [64]. In a similar manner, MAPE and R^2^ are also scaled independent parameters; therefore, they can be used to compare model performance with existing methods implemented over data with different scales [64]. The performance was analysed for all six IAQ parameters, and the graphical plots of NRMSE for all six response variables are shown in Figure S2 (Supplementary Materials).

The performance evaluation tests were conducted on both training and validation data. Observations ensure that the model does not overfit, even after high variance (Table 2) in the field data, as prediction performance was comparable in both cases. The NRMSE values for all six cases for training and validation data are shown in Table 5. For PM_10_ prediction, the PSO optimized model provided NRMSE of 0.8046, which was further improved with Pattern Search-based model tuning to 0.6679. The NRMSE prediction performance graph for PM_10_ in Figure S2a (Supplementary Materials) also indicates a close association between validation data and predicted output. In a similar manner, NRMSE of ADFIST for PM_2.5_, CO_2_, tVOC, CO and NO_2_ was recorded to be 0.6679, 0.6218, 0.1077, 0.2585, 0.0667 and 0.0635, respectively. Moreover, the numerical values for all performance indicators at two different stages of the proposed ADFIST are provided in Table 6. It can be observed that the proposed model shows efficient performance for predicting all different IAQ parameter conditions. Although the combination of hierarchical approach with optimized rule base helped to reduce the computational complexity of the forecasting system by a considerable level, the computational cost needs slight improvement. The proposed ADFIST system took 1 h 38 min for training; the evaluation time was recorded around 3 min 56 s. However, it is a great improvement to the simple FIS system; several other models exist in the literature that are computationally less expensive. Among the many advantages of the proposed ADFIST system, higher computational cost is a main limitation that requires further improvement.

A direct comparison of results obtained from the given experimental analysis with existing studies in the literature is limited due to several factors [76]. The first important aspect is the difference in datasets used for model training and the type of methods [76,77]. Several researchers in the past have used IAQ and thermal comfort datasets from meteorological websites and air pollution control boards [78,79]. However, very few considered data from real-time scenarios that include concentration measurement from sensor units [21,80]. Moreover, the type of sensors used to measure field pollutant concentrations also makes a considerable difference in the prediction outcomes [81,82].

High-quality sensors can provide more reliable field measurements, but the cost involved in system design and installation turns out to be very high [23]. On the other side, the low-cost sensors are likely to experience calibration issues, and the measured parameter concentrations may show high variance [24]. The authors have also used low-cost sensors for field data measurements, and the statistical information about data is already provided in Table 2. It is clear from the last row of Table 2 that measured parameters show high variance, especially in the case of PM_10_, PM_2.5,_ and CO_2_. Moreover, the min, max, and mean values of the parameter concentrations also vary from one geographical region to another. Therefore, it is difficult to compare an analysis performed on one dataset using a specific type of method with an analysis performed on other datasets using a different method.

The variation in forecasting performance can also be observed based on the performance indication parameter selection and the pre-processing data methods used by researchers [65,83,84]. Several researchers prefer using scaling methods (normalization or standardization) for field data due to differences in the scales and units of the measured parameters [85,86,87]. This technique is necessary while processing time-series data using neural networks, deep learning, and machine learning-based methods [88]. However, in this paper, the authors worked on FIS, where field data is processed without scaling. The main reason to use data without scaling is to design a FIS knowledgebase by focusing on actual parameter concentration ranges as defined by air pollution experts. The field information can be further mapped to the fuzzy values ranging between 0 and 1 through respective membership functions that are assigned as per pollutant concentration ranges. Additionally, different studies available in the literature use unique performance indicators. Numerous researchers worked on RMSE and MSE parameters (scale dependent), preferably when they have scaled the input data before model training [89,90,91]. Others preferred using MAE, correlation coefficient (R), and MAPE (%) for analysing model performance [92,93,94,95]. Another challenge for comparison with existing studies was to identify works that used similar parameters for IAQ analysis. The selection of parameters for IAQ analysis usually varies as per the common sources of pollution, geographical conditions, and lifestyle of residents in the target area. Therefore, only a limited number of studies can be used for comparison with the proposed model.

The performance of the proposed ADFIST system was compared with the existing studies in the literature (Table 7). As no other study with similar kinds of parameters was available, the authors compared the performance with different works published in the past to provide an analysis relevant to all six IAQ prediction parameters under consideration. As the type of air pollution parameters and the performance indicators used by existing studies may be different, the authors selected only similar kinds of parameters and indicators for comparison out of selected papers. The first study added for comparison in Table 7 is proposed by [96]. The authors in this study [96] used SO_2_, NO_2_, CO, O_3,_ and PM_10_ for ambient air pollution analysis, and the data were collected from State Pollution Control Board, West Bengal, India. However, for comparison, only three relevant parameters were considered that are similar to parameters used in the current study, and the comparison was made on the basis of NMSE and R_2_ values.

Prasad et al. used normalized ambient air pollution and meteorology data for analysis, and they used Adaptive Neuro-Fuzzy Inference System (ANFIS) model for forecasting respective pollutant concentrations [96]. In the comparative study, multi-collinearity tests eliminated the redundant input variables, and a forward selection method was utilized for choosing different subsets of input variables with an aim to reduce the computational cost and time of the method. However, in this study, the authors used PSO and pattern search algorithm to reduce the computational time and cost of the proposed ADFIST system. In this case, PSO helped to generate the most relevant rule base for the DFIST training in lesser time, and the pattern search algorithm eliminated the redundant feature combinations and rules during training while reducing the computational cost. The adaptive rule base generated with the combination of PSO and pattern search algorithm at each stage of ADFIST is already given in Table 4. As shown in the comparison shown in Table 7, the existing study provided R^2^ = 0.71, NMSE = 0.23 for PM_10_ forecasting, whereas the proposed method presents R^2^ = 0.8172, and NMSE = 0.1822. For CO forecasting, the existed authors presented R^2^ = 0.77, NMSE = 0.33; however, the proposed method provided R^2^ = 0.6824 and NMSE = 0.0667. Similarly, for NO_2_, the existing method provided R^2^ = 0.85 and NMSE = 0.00; however, the proposed method provided R^2^ = 0.7201 and NMSE = 0.2799.

The performance of the proposed method was observed to be better compared to the existing studies [96,97] for PM_10_ forecasting. However, in the case of NO_2_ and CO forecasting, the results are required to be improved. As the R^2^ parameter is highly affected by variance among dependent and independent variables, it is difficult to rely on this parameter specifically for performance assessment, especially when different datasets are involved in the study [99]. However, the authors in the future are also planning to make further analyses on feature importance and relevant combinations at different stages of ADFIST so that prediction accuracy for these response variables can be improved.

Another study used for comparison of model performance was published by [97]. The authors in this study used an Adaptive Neuro-Fuzzy weighted extreme learning machine (ANFIS-WELM) for forecasting concentrations of CO, NO, PM_2.5_ and PM_10_. The field pollutant concentration data of Dotong Railway station was collected from Environment Protection Administration in Northern Taiwan. The authors used the unique combination of ANFIS and WELM to improve the prediction accuracy and generalization ability of the proposed model. The main contribution of [97] was that the authors provided separate and detailed analyses on multiscale air pollutant concentration with hourly forecasts. It was observed that the proposed method performed well for short-term predictions as compared to long-term predictions. The performance of the existing study was compared to the proposed method on the basis of three important pollutants (CO, PM_2.5,_ and PM_10_), considering MAPE as a common performance indicator. The existing study provided MAPE = 22.13%, 30.848% and 7.1250% for CO, PM_2.5_ and PM_10_, respectively; however, the proposed study provided MAPE = 0.0500%, 4.428% and 3.609% for CO, PM_2.5_ and PM_10_, respectively.

In [98], the authors worked on CO_2_, tVOC, and HCHO parameters measured from five different rooms on a school campus at the University of Singapore. Authors in this paper reported Support Vector Machine (SVM) as the best method for forecasting CO_2_ and tVOC concentrations. The analysis also reports SVM as the optimal data mining algorithm to receive the most accurate and reliable results, leading to high-precision predictions. The best R^2^ values achieved by the existing study for CO_2_ and tVOC are 0.9883 and 0.9636, respectively. However, the MAPE (%) values for CO_2_ and tVOC are 1.59 and 2.30, respectively. The proposed method achieved comparatively lesser performance for these pollutants in terms of R^2^ with values equal to 0.8811 and 0.9017 for tVOC and CO_2_, respectively. Table 5 shows NRMSE = 0.1077 and NMSE = 0.0983 for CO_2_ prediction, which indicates reliable performance for forecasting. However, the main reason behind poor performance in terms of R^2^ for CO_2_ prediction is that this performance indicator is highly affected by variance in data. Table 2 already shows a very high variance in the field measurements of CO_2,_ which is automatically reflected in the R^2^ parameter value. Moreover, as seen from Table 4, unlike other response variables, PSO convergence was not achieved up to 150 iterations for CO_2_. Therefore, the performance can be further enhanced by increasing the number of iterations at the first stage. It can improve the rule learning process while enhancing the model’s ability to provide more accurate predictions. Furthermore, MAPE (%) performance achieved with the proposed method for CO_2_ was 0.0666. However, for tVOC, the exact value of MAPE could not be received due to zeros present in the actual measurements.

### Model Validation with Online GAMS Dataset

The results show that the proposed ADFIST model ensures satisfactory performance for forecasting real-time IAQ data. However, it is critical to test the proposed unique approach against another dataset to ensure that it does not follow data-dependent behaviour. It was not possible to find another benchmark dataset with similar kinds of parameters. Therefore, in order to validate the proposed approach, the authors considered the GAMS dataset that is available online with six relevant IAQ and thermal comfort parameters: PM_10_, PM_2.5_, CO_2_, VOC, temperature, and humidity [100]. The total number of samples (from 21 November 2016 to 28 March 2017) available in this online dataset are 1,35,100 with per minute data collection from the field environment. The data contained 0.04% missing rows that were filled using the mean imputation method, as mentioned in Section 2. After mean-hour conversion, the remaining number of samples was 3058. The statistical information of the pre-processed online dataset is provided in Table 8.

The data was further divided into a training set and validation set using a 20% holdout approach with k-fold cross-validation, as discussed in Section 2. The number of samples used for model training was 2446, whereas 612 samples were used for validation. In order to accommodate five input variables and one response variable, the ADFIST model used four—two input and one output—FIS modules. The input parameter combination was followed as per the correlation analysis already discussed in Section 3. The performance of ADFIST considering all four IAQ parameters of the GAMS dataset are provided in Table 9. In order to predict the concentration of the PM_10_ parameter, ADFIST used rules. As the model had a lesser number of parameters as compared to the previously discussed version, the number of iterations at the first stage for PSO rule learning were limited to 100, and the number of iterations used at the second stage were 80. However, PSO rule learning presented fast convergence in few cases. The NRMSE plots for all four response variables are given in Figure S3 (Supplementary Materials).

From the above analysis, it can be observed that the proposed approach is not data dependent. Rather, this approach can be used for handling a variety of field IAQ data to provide real-time assessment and forecasting on parameter concentrations. Table 6, Table 7 and Table 8 show satisfactory forecasting performance for the proposed ADFIST model. However, the proposed system also has few limitations. The main problem is the use of low-cost sensor units. Although the used sensors are factory calibrated and reliability tests are also conducted before installation, the field data still show considerable variance and the presence of outliers. Moreover, the study is based on only six IAQ parameters and two thermal comfort parameters that are relevant to the selected geographical area.

In the future, the authors are also planning to analyse the performance of the proposed ADFIST system on other relevant pollutant concentrations received from different geographical conditions. Furthermore, it is also necessary to integrate the proposed model with an online portal or mobile application that could generate real-time alerts to guide building occupants regarding critical scenarios. Consequently, the authors are planning to develop a standalone system that could provide real-time IAQ assessment, forecasting, and prior alerts relevant to important pollutant concentrations. These systems can be more useful for improving public health and wellbeing in a building environment.

## 5. Conclusions

The main contribution of this paper is the unique integration of correlation based DFIST, optimized with PSO and pattern search at two different stages. The proposed ADFIST receives field inputs from an IoT-based hardware module, installed in a rural village of India. It measures eight different IAQ and thermal comfort parameters from the target environment. A total 42051 samples were collected with real-time monitoring; however, after pre-processing and mean-hour conversion, the model was trained using 2956 samples and validation tests were conducted on 739 samples.

The proposed ADFIST model process input features were based on the correlation with the target variable. The membership functions are defined as per the pollutant concentration ranges defined by field experts; however, the knowledge base is generated with the help of a global optimization algorithm—PSO. The model performance was further enhanced with the input–output and rule tuning, using the pattern search algorithm. The proposed model shows reliable prediction performance for all six IAQ parameters with the optimized knowledge base. One important concern with this system is that it makes use of hierarchal structure for evaluating field variables. If the same method is used for the higher number of variables, the network may become too complex to handle. However, for a limited number of input parameters, this system can perform well while ensuring lesser computational complexity. This is because there is no need to process all input parameters at a single stage that may otherwise lead to a highly complicated rule base, while increasing the computational complexity of the network. When the inputs are arranged in hierarchal order, the system shows enhanced performance, even with a limited number of rules.

In future work, the methodology can be further enhanced to accommodate a wide range of environmental variables to ensure a real-time alert system for the end-users. However, one of the prime challenges for authors is to reduce the computational costs of the proposed ADFIST system. The main goal is to design an efficient, cost-effective, and easy-to-use standalone system that could be installed in urban as well as rural buildings to perform real-time assessment of pollutant concentrations. Furthermore, a mobile app or web portal-based alerts can be created to help the building occupants to avoid the critical consequences associated with pollutant concentration levels.

## Figures and Tables

**Table 1:** Manufacturer specifications for sensors used to design IoT based hardware module.

Sensor Name	Manufacturer	Type of Sensor	Measurement Parameter	Typical Range	Cost of Sensor/Unit (US Dollars)
CCS811	SparkFun	Digital Sensor	CO_2_, tVOC	0–1187 ppb (tVOC); 400–8192 ppm (CO_2_)	$24.28
SDS011	Nova Fitness	Laser Sensor	PM_10_, PM_2.5_	0.0–999.9 μg/m^3^	$31.70
Grove—Air quality sensor v1.3—MP503	Seed Studio	Digital Sensor	CO, NO_2_	NA	$12.82
DHT11	Aosong MPN	Negative Temperature Coefficient (NTC)	Temperature, Humidity	0 °C to 50 °C; 20% to 90%	$1.35

**Table 2:** Statistical information about measured and pre-processed field parameters.

	Temp	Hum	PM_25_	PM_10_	CO	NO_2_	CO_2_	tVOC
Count	3695.00	3695.00	3695.00	3695.00	3695.00	3695.00	3695.00	3695.00
Mean	29.900	53.921	124.117	144.724	67.477	67.088	745.133	52.780
Std	1.915	9.841	200.603	229.093	8.103	8.156	249.922	39.707
Min	22.100	23.750	1.736	2.337	38.000	36.000	400.000	0.000
25%	29.690	48.916	8.608	13.051	62.833	62.333	566.090	24.784
50%	29.900	53.921	20.381	30.541	67.477	67.088	735.166	50.416
75%	31.250	60.818	124.117	144.724	72.916	72.727	770.606	55.958
Max	33.218	79.000	999.90	1378.90	93.500	93.714	1544.00	203.00
Variance	3.668	96.825	40,230.93	52,469.67	65.644	66.503	62,444.47	1576.28

**Table 3:** Input feature arrangements at ADFIST system with respect to the selected response variable.

FIS1		FIS2		FIS4		FIS6	Response Variable
Input 1	Input 2	Input 1	Input 2	Input 1	Input 2	Input 1	-
Temp	PM_2.5_	CO_2_	tVOC	CO	NO_2_	Hum	PM_10_
Hum	PM_10_	CO_2_	tVOC	CO	NO_2_	Temp	PM_2.5_
Hum	PM_10_	PM_2.5_	tVOC	CO	NO_2_	Temp	CO_2_
Temp	NO_2_	CO_2_	tVOC	PM_2.5_	PM_10_	Hum	CO
Temp	CO_2_	PM_10_	PM_2.5_	CO	NO_2_	Hum	tVOC
Temp	CO	CO_2_	tVOC	PM_2.5_	PM_10_	Hum	NO_2_

**Table 4:** Adaptive knowledge base of proposed ADFIST model.

Number of Iterations (PSO Rule Learning + Pattern Search Tuning)	Number of Rules at Different Stages of ADFIST						Total Number of Rules	Response Variable
-	FIS1	FIS2	FIS3	FIS4	FIS5	FIS6	-	-
116 + 100	16	21	22	23	17	12	111	PM_10_
145 + 100	12	19	17	21	20	10	99	PM_2.5_
150 + 100	15	22	23	20	22	16	118	CO_2_
133 + 100	14	22	22	20	22	13	113	tVOC
136 + 100	13	20	22	22	22	14	113	CO
140 + 100	13	20	24	23	20	15	115	NO_2_

**Table 5:** ADFIST performance evaluation on training and validation data.

Response Variable	NRMSE (Training Data)	NRMSE (Validation Data)
PM_10_	0.7000	0.6679
PM_2.5_	0.6371	0.6218
CO_2_	0.1018	0.1077
tVOC	0.2627	0.2585
CO	0.0677	0.0667
NO_2_	0.0643	0.0635

**Table 6:** NRMSE performance of ADFIST after PSO based rule learning and pattern search-based tuning.

Performance Indicator	Response Variable	ADFIST	
		DFIST + PSO	DFIST + PSO + Pattern Search
NRMSE	PM_10_	0.8046	0.6679
	PM_2.5_	0.7334	0.6218
	CO_2_	0.1811	0.1077
	tVOC	0.3741	0.2585
	CO	0.0800	0.0667
	NO_2_	0.0863	0.0635
NMSE	PM_10_	0.2645	0.1822
	PM_2.5_	0.2071	0.1489
	CO_2_	0.2783	0.0983
	tVOC	0.2489	0.1189
	CO	0.4574	0.3176
	NO_2_	0.5162	0.2799
R^2^	PM_10_	0.7355	0.8178
	PM_2.5_	0.7929	0.8511
	CO_2_	0.7217	0.9017
	tVOC	0.7511	0.8811
	CO	0.5426	0.6824
	NO_2_	0.4838	0.7201
MAPE (%)	PM_10_	4.409	3.609
	PM_2.5_	4.657	4.428
	CO_2_	0.0904	0.0666
	tVOC	Inf	Inf
	CO	0.0609	0.0500
	NO_2_	0.0669	0.0457

**Table 7:** Comparison of proposed Optimized DFIST model with existing studies in the literature.

Method	Dataset	Normalization	Response Variable	Performance	Ref
ANFIS	State Pollution Control Board, West Bengal, India (Ambient Air Pollution)	Yes	PM_10_	R^2^ = 0.71 NMSE = 0.23	[96]
			CO	R^2^ = 0.77 NMSE = 0.33	
			NO_2_	R^2^ = 0.85 NMSE = 0.00	
ANFIS-WELM	Environmental Protection Administration, Northern Taiwan (Railway Station)	Yes	CO	MAPE = 22.13%	[97]
			PM_10_	MAPE = 7.1250%	
			PM_2.5_	MAPE = 30.948%	
SVM	INNOVA—Multipoint sampler and multi-gas monitor (School Campus)	NA	CO_2_	R^2^ = 0.9883 MAPE = 1.59%	[98]
			tVOC	R^2^ = 0.9636 MAPE = 2.01%	
Proposed Method (ADFIST)	IoT based Low-Cost Sensor Hardware (Rural Home)	No	PM_10_	R^2^ = 0.8178 NMSE = 0.1822 MAPE = 3.609%	-
			PM_2.5_	MAPE = 4.428%	
			CO_2_	R^2^ = 0.9017 MAPE = 0.0666%	
			CO	R^2^ = 0.6824 NMSE = 0.0667 MAPE = 0.0500%	
			tVOC	R^2^ = 0.8811 MAPE = Inf	
			NO_2_	R^2^ = 0.7201 NMSE = 0.2799	

**Table 8:** Statistical information of pre-processed GAMS Dataset.

	CO_2_	Hum	PM_10_	PM_2.5_	Temp	VOC
Count	3058.00	3058.00	3058.00	3058.00	3058.00	3058.00
Mean	716.030509	38.422101	17.378770	15.826833	23.016499	0.117204
Std	402.048356	5.445556	12.662556	11.894725	2.058361	0.082843
Min	372.633333	22.140000	0.833333	0.733333	18.116818	0.062000
25%	433.284545	34.766833	8.155833	7.266118	21.482593	0.064250
50%	501.388889	38.422101	13.807500	12.301667	22.982167	0.079508
75%	894.812500	41.974500	22.826147	20.848873	24.726168	0.138531
Max	2570.409091	68.351538	84.356250	72.896774	27.914815	0.695500
Variance	161,590.0219	29.644378	160.287883	141.438205	4.235465	0.006861

**Table 9:** ADFIST performance analysis with GAMS dataset.

Response Variable	Number of Rules	Number of Iterations	NRMSE	NMSE	R^2^	MAPE (%)
PM_10_	70	100 + 80	0.3781	0.2655	0.7345	0.5503
PM_2.5_	74	100 + 56	0.4799	0.3807	0.6193	0.9204
CO_2_	69	93 + 80	0.3316	0.3258	0.6742	0.2422
VOC	65	96 + 80	0.6392	0.3473	0.6527	0.5374

## Data Availability

Not applicable.

## Supplementary Materials

The following supporting information can be downloaded at: https://www.mdpi.com/article/10.3390/s22031008/s1, Figure S1: Boxplot representation of monitored IAQ parameter concentrations; Figure S2: NRMSE based prediction performance of ADFIST for (a) PM10 (b) PM2.5 (c) CO_2_ (d) tVOC (e) CO (f) NO_2_; Figure S3: NRMSE Performance of ADFIST on Gams Dataset (a) PM10, (b) PM2.5, (c) CO_2_ and (d) VOC; Algorithm S1: General Algorithm for PSO; Algorithm S2: General Algorithm for Pattern Search.
