# Supplementary material for: ADFIST: Adaptive Dynamic Fuzzy Inference System Tree Driven by Optimized Knowledge Base for Indoor Air Quality Assessment
Source: Sensors (Basel). 2022 Jan 28;22(3):1008. doi: 10.3390/s22031008 (PMC8838659; doi:10.3390/s22031008)
Supplement: Supplementary file 1 [file sensors-22-01008-s001.zip › sensors-1485008-supplementary.pdf]

Supplementary Materials

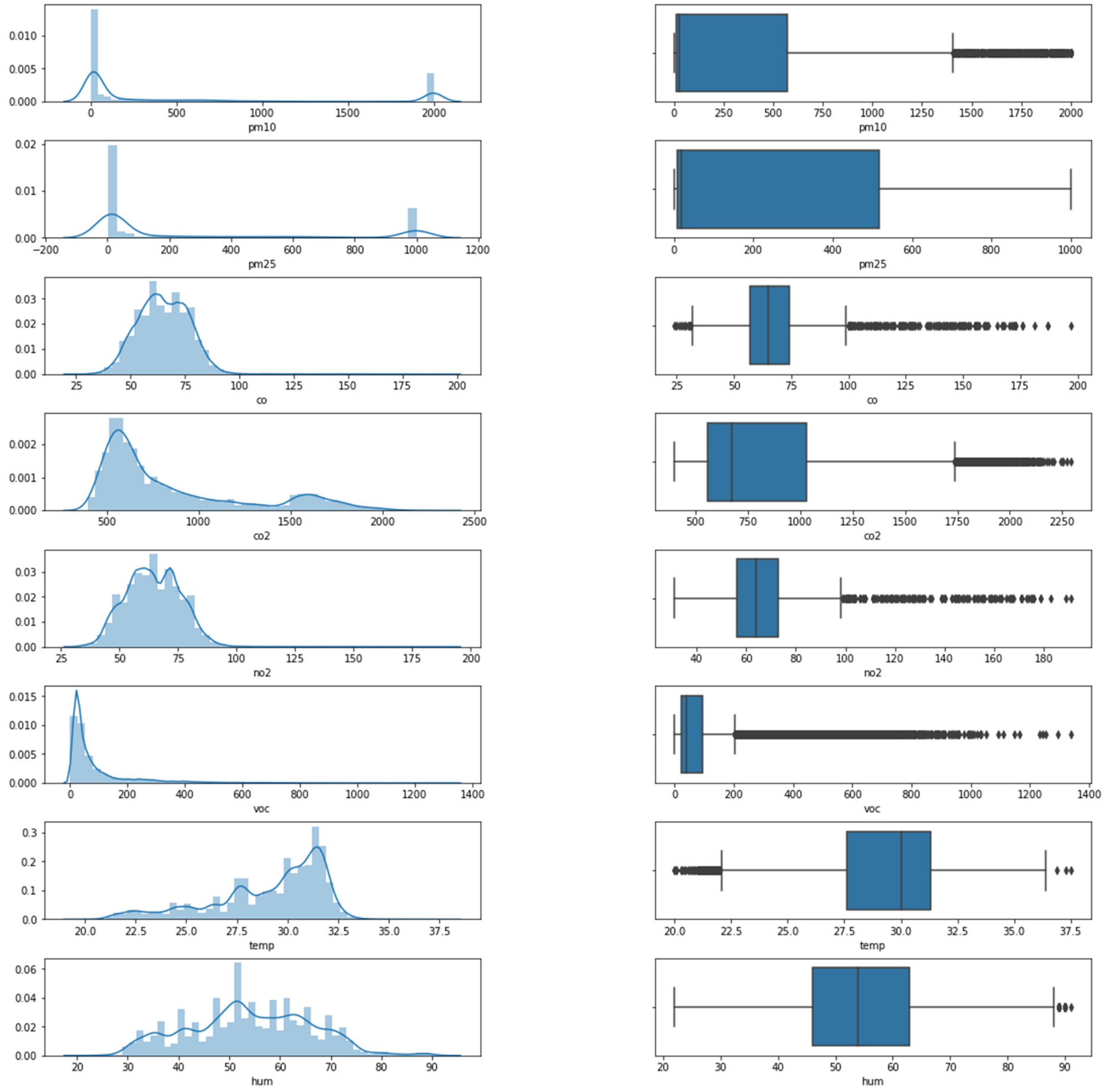

Figure S1. Boxplot representation of monitored IAQ parameter concentrations.

Algorithm S1: General Algorithm for PSO

procedure PSO
for each particle p in swarm S do
Initialize parameters
Evaluate fitness F_i for particle
end for
evaluate p_{best}, g_{best}
for i = 1 to max_iterations do
for each particle p in swarm S evaluate
$$V_i(t+1) = wV_i(t) + c_1 r_1 \left(P_{pbest}(t) - P_i(t)\right) + c_2 r_2 (P_{gbest}(t) - P_i(t))$$

10: $P_i(t+1) = P_i(t) + V_i(t+1)$
11: **end for**
12: Update p_{best}, g_{best}
13: Choose best value of fitness as solution
14: End if termination condition met
15: **end for**
16: **end procedure**

Algorithm S2: General Algorithm for Pattern Search

1: **For pattern search procedure**
2: **Initialize**
3: x(0)
4: Reduction factor $\alpha > 1$
5: Increment *Δ(i)*
6: Termination criterion ε.
7: Set *k = 0; x(k)* is base point of search.
8: **If** a successful movement occurs
9: set *x(k) = x* and switch to step 19
10: else
11: go to step 13
12: **end if**
13: **If** $||\Delta|| < \varepsilon$
14: terminate
15: else
16: set *Δ(i) = Δ(i)/ α* and go to step 7.
17: **end if**
18: Set *k = k+1;*
19: move *x(k+1) =*
x(k)+[x(k) − x(k − 1)]
20: **If** *f[x(k+1)] < f[x(k)]*
21: go to step 19
22: else
23: move to step 13.
24: **end if**
25: **end procedure**

Optimized Rule Base for FIS1: 1x16 Array

1 “temp==mf3 & pm25==mf5 => output1=mf5 (1)”
2 “temp==mf1 & pm25==mf4 => output1=mf2 (1)”
3 “pm25==mf2 => output1=mf1 (1)”
4 “temp==mf1 & pm25==mf5 => output1=mf4 (1)”
5 “pm25==mf4 => output1=mf4 (1)”
6 “temp==mf1 & pm25==mf3 => output1=mf3 (1)”
7 “temp==mf1 & pm25==mf2 => output1=mf5 (1)”
8 “pm25==mf3 => output1=mf4 (1)”
9 “temp==mf1 => output1=mf2 (1)”
10 “temp==mf3 => output1=mf1 (1)”
11 “temp==mf3 & pm25==mf3 => output1=mf4 (1)”
12 “temp==mf2 & pm25==mf1 => output1=mf2 (1)”
13 “temp==mf3 & pm25==mf2 => output1=mf1 (1)”
14 “temp==mf1 & pm25==mf1 => output1=mf1 (1)”

15 “temp==mf2 & pm25==mf3 => output1=mf1 (1)”
16 “pm25==mf1 => output1=mf2 (1)”

Optimized Rule Base for FIS2: 1x21 Array
1 “co2==mf5 & voc==mf1 => output1=mf1 (1)”
2 “co2==mf1 & voc==mf4 => output1=mf4 (1)”
3 “co2==mf3 & voc==mf3 => output1=mf3 (1)”
4 “co2==mf4 & voc==mf2 => output1=mf1 (1)”
5 “co2==mf5 & voc==mf5 => output1=mf3 (1)”
6 “co2==mf2 & voc==mf1 => output1=mf2 (1)”
7 “voc==mf1 => output1=mf2 (1)”
8 “voc==mf3 => output1=mf3 (1)”
9 “co2==mf1 & voc==mf1 => output1=mf5 (1)”
10 “co2==mf5 & voc==mf2 => output1=mf1 (1)”
11 “co2==mf3 & voc==mf1 => output1=mf1 (1)”
12 “co2==mf4 & voc==mf5 => output1=mf4 (1)”
13 “voc==mf5 => output1=mf5 (1)”
14 “co2==mf2 & voc==mf3 => output1=mf2 (1)”
15 “co2==mf2 & voc==mf5 => output1=mf2 (1)”
16 “co2==mf2 => output1=mf4 (1)”
17 “co2==mf3 & voc==mf5 => output1=mf2 (1)”
18 “co2==mf3 => output1=mf5 (1)”
19 “co2==mf3 & voc==mf2 => output1=mf1 (1)”
20 “co2==mf5 & voc==mf3 => output1=mf1 (1)”
21 “co2==mf5 & voc==mf4 => output1=mf3 (1)”

Optimized Rule Base for FIS3: 1x22 Array
1 “input1==mf4 & input2==mf3 => output1=mf5 (1)”
2 “input1==mf3 => output1=mf3 (1)”
3 “input1==mf2 & input2==mf4 => output1=mf2 (1)”
4 “input2==mf4 => output1=mf5 (1)”
5 “input2==mf5 => output1=mf1 (1)”
6 “input1==mf4 & input2==mf4 => output1=mf3 (1)”
7 “input1==mf1 => output1=mf3 (1)”
8 “input1==mf1 & input2==mf2 => output1=mf1 (1)”
9 “input1==mf1 & input2==mf5 => output1=mf1 (1)”
10 “input1==mf4 & input2==mf2 => output1=mf4 (1)”
11 “input1==mf5 => output1=mf5 (1)”
12 “input1==mf4 & input2==mf1 => output1=mf2 (1)”
13 “input2==mf2 => output1=mf4 (1)”
14 “input1==mf3 & input2==mf3 => output1=mf5 (1)”
15 “input1==mf5 & input2==mf2 => output1=mf5 (1)”
16 “input1==mf5 & input2==mf4 => output1=mf4 (1)”
17 “input2==mf1 => output1=mf1 (1)”
18 “input1==mf5 & input2==mf1 => output1=mf1 (1)”
19 “input1==mf2 & input2==mf3 => output1=mf1 (1)”
20 “input1==mf2 & input2==mf2 => output1=mf4 (1)”
21 “input1==mf5 & input2==mf3 => output1=mf1 (1)”
22 “input2==mf3 => output1=mf4 (1)”

Optimized Rule Base for FIS4: 1x23 Array
1 “co==mf5 & no2==mf3 => output1=mf1 (1)”
2 “co==mf2 & no2==mf5 => output1=mf4 (1)”
3 “co==mf3 => output1=mf5 (1)”

4 “co==mf5 => output1=mf4 (1)”
5 “co==mf3 & no2==mf3 => output1=mf2 (1)”
6 “co==mf4 & no2==mf5 => output1=mf4 (1)”
7 “co==mf1 & no2==mf2 => output1=mf3 (1)”
8 “co==mf1 & no2==mf1 => output1=mf3 (1)”
9 “co==mf3 & no2==mf2 => output1=mf3 (1)”
10 “co==mf2 & no2==mf3 => output1=mf1 (1)”
11 “no2==mf2 => output1=mf4 (1)”
12 “co==mf1 => output1=mf1 (1)”
13 “co==mf5 & no2==mf4 => output1=mf1 (1)”
14 “no2==mf5 => output1=mf1 (1)”
15 “co==mf5 & no2==mf1 => output1=mf4 (1)”
16 “co==mf4 => output1=mf1 (1)”
17 “co==mf3 & no2==mf1 => output1=mf1 (1)”
18 “no2==mf4 => output1=mf4 (1)”
19 “co==mf4 & no2==mf3 => output1=mf4 (1)”
20 “co==mf2 & no2==mf4 => output1=mf1 (1)”
21 “co==mf2 & no2==mf2 => output1=mf1 (1)”
22 “co==mf1 & no2==mf3 => output1=mf3 (1)”
23 “co==mf4 & no2==mf1 => output1=mf2 (1)”

Optimized Rule Base for FIS5: 1x17 Array
1 “input1==mf5 & input2==mf3 => output1=mf1 (1)”
2 “input1==mf4 & input2==mf5 => output1=mf5 (1)”
3 “input1==mf3 & input2==mf5 => output1=mf1 (1)”
4 “input1==mf2 & input2==mf5 => output1=mf5 (1)”
5 “input1==mf5 & input2==mf5 => output1=mf1 (1)”
6 “input1==mf1 & input2==mf2 => output1=mf4 (1)”
7 “input1==mf1 & input2==mf1 => output1=mf5 (1)”
8 “input1==mf1 & input2==mf5 => output1=mf2 (1)”
9 “input1==mf5 & input2==mf2 => output1=mf1 (1)”
10 “input1==mf5 => output1=mf5 (1)”
11 “input1==mf1 & input2==mf4 => output1=mf1 (1)”
12 “input1==mf3 & input2==mf3 => output1=mf3 (1)”
13 “input2==mf4 => output1=mf1 (1)”
14 “input1==mf4 => output1=mf3 (1)”
15 “input1==mf3 & input2==mf4 => output1=mf1 (1)”
16 “input1==mf2 & input2==mf1 => output1=mf1 (1)”
17 “input1==mf5 & input2==mf4 => output1=mf1 (1)”

Optimized Rule Base for FIS6: 1x12 Array
1 “input1==mf4 & hum==mf3 => pm10=mf1 (1)”
2 “input1==mf3 & hum==mf2 => pm10=mf1 (1)”
3 “input1==mf1 & hum==mf1 => pm10=mf2 (1)”
4 “input1==mf1 & hum==mf3 => pm10=mf1 (1)”
5 “input1==mf5 => pm10=mf1 (1)”
6 “input1==mf4 & hum==mf2 => pm10=mf2 (1)”
7 “input1==mf2 & hum==mf1 => pm10=mf1 (1)”
8 “input1==mf4 & hum==mf1 => pm10=mf1 (1)”
9 “input1==mf2 => pm10=mf3 (1)”
10 “input1==mf5 & hum==mf2 => pm10=mf5 (1)”
11 “input1==mf2 & hum==mf3 => pm10=mf5 (1)”
12 “input1==mf3 => pm10=mf1 (1)”

Where membership functions represent:
mf1 = Good/Low
mf2 = Moderate
mf3 = Unhealthy/High
mf4 = Poor
mf5 = Hazardous

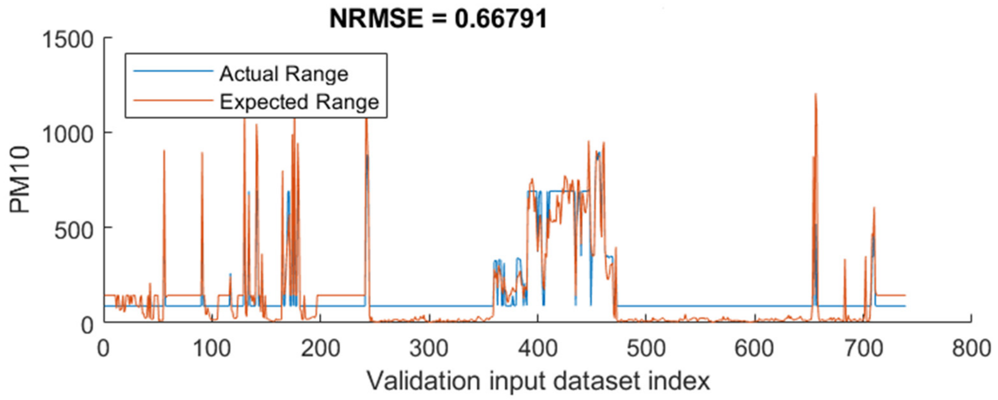

(**a**)

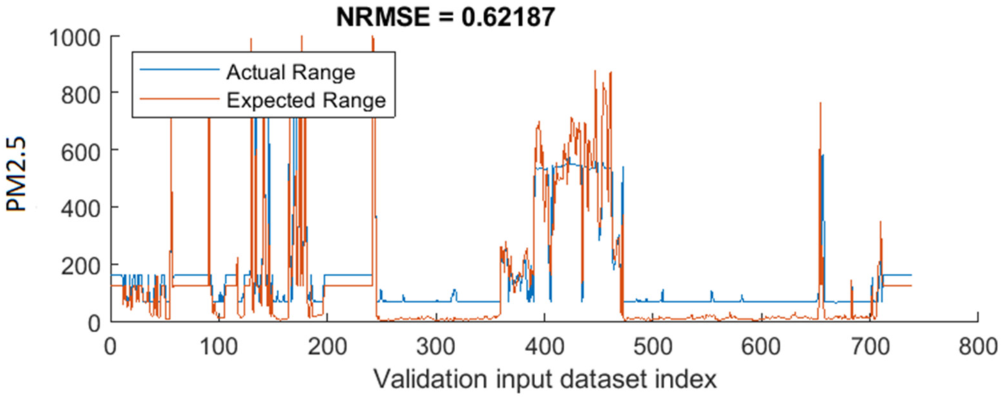

(**b**)

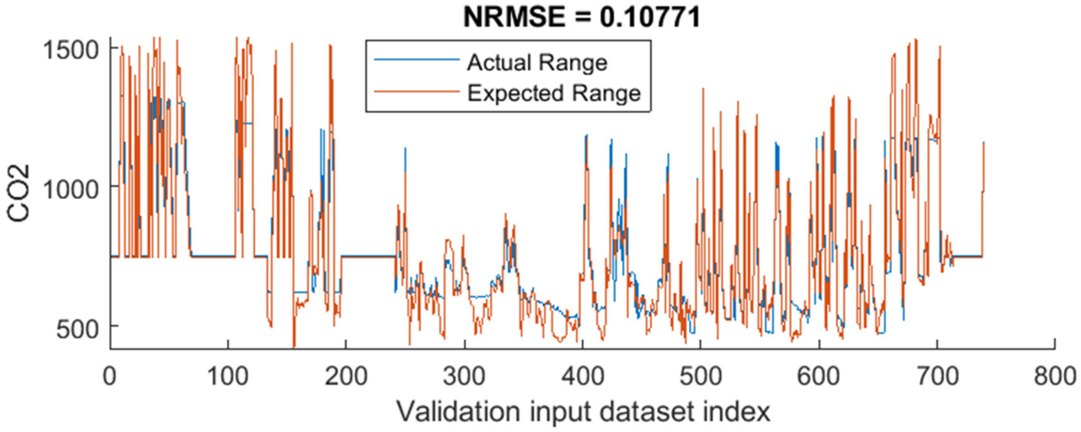

(**c**)

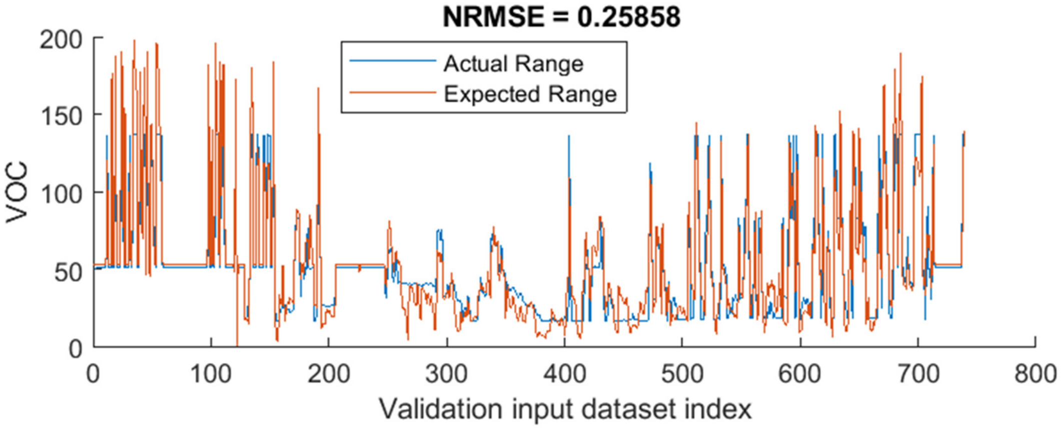

(**d**)

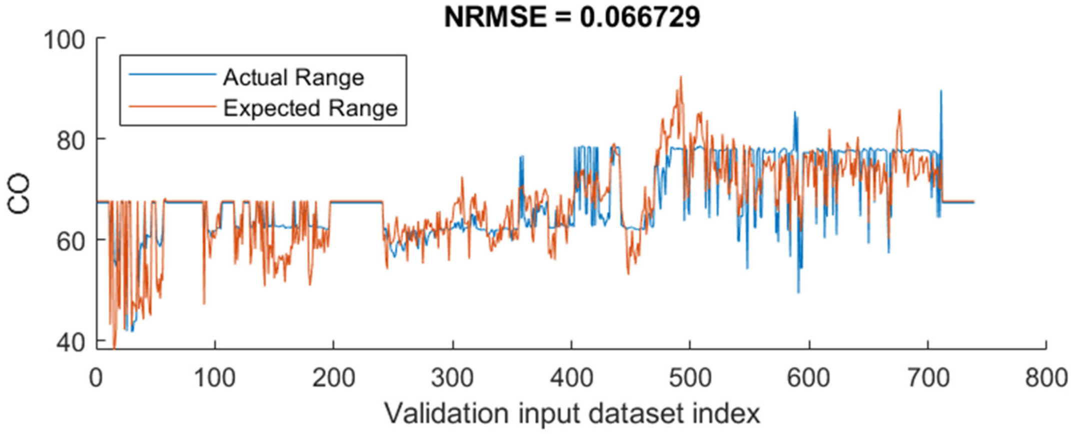

(**e**)

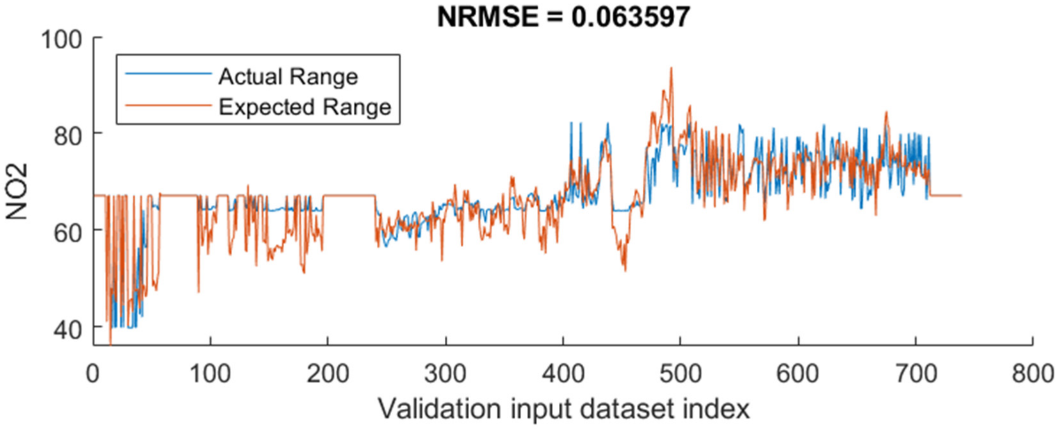

(**f**)

Figure S2. NRMSE based prediction performance of ADFIST for (**a**) PM_{10} (**b**) $PM_{2.5}$ (**c**) CO_2 (**d**) tVOC (**e**) CO (**f**) NO_2.

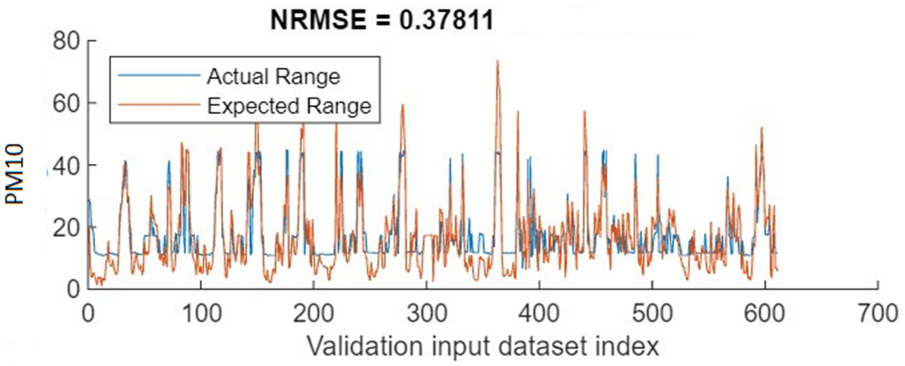
NRMSE = 0.37811
Actual Range
Expected Range
PM10
Validation input dataset index

(**a**)

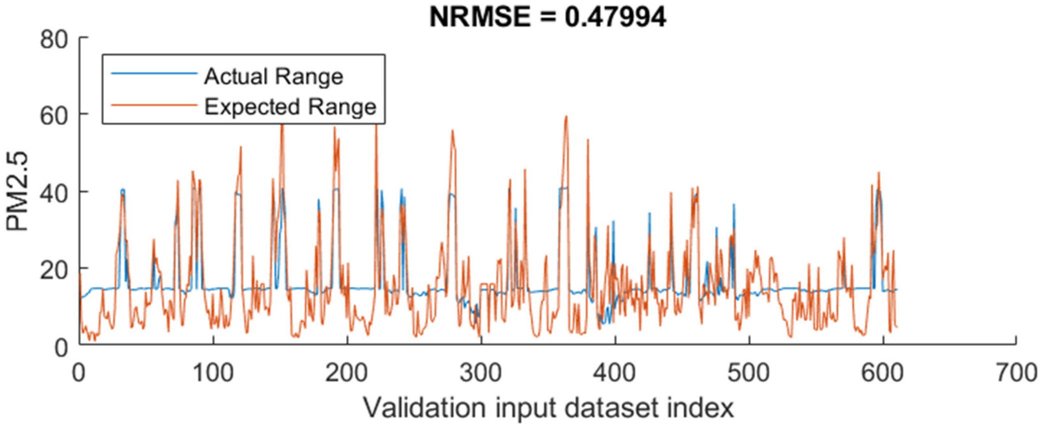
NRMSE = 0.47994
Actual Range
Expected Range
PM2.5
Validation input dataset index

(**b**)

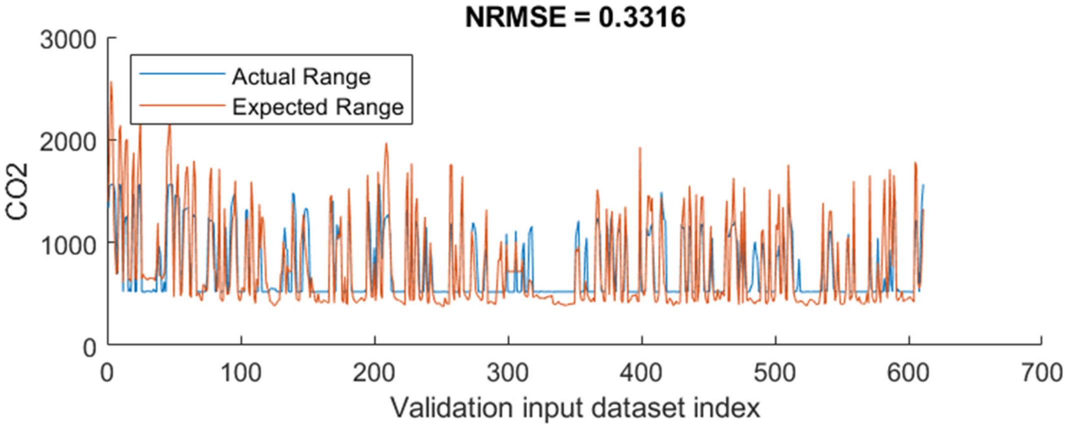
NRMSE = 0.3316
Actual Range
Expected Range
CO2
Validation input dataset index

(**c**)

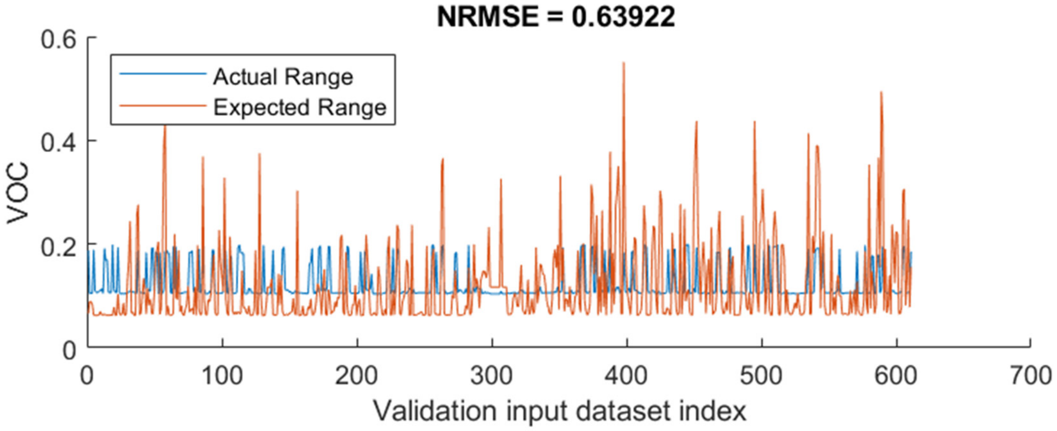
NRMSE = 0.63922
Actual Range
Expected Range
VOC
Validation input dataset index

(**d**)

Figure S3 NRMSE Performance of ADFIST on Gams Dataset (**a**) PM_{10}, (**b**) $PM_{2.5}$, (**c**) CO_2 and (**d**) VOC.
